# Biomechanical study using the finite element analysis of a new method for fixation of coronal fracture of the femoral condyle

**DOI:** 10.1016/j.jor.2026.04.006

**Published:** 2026-04-19

**Authors:** Celso Júnio Aguiar Mendonça, Gabriel Olesczuk, Matheus Fernandes Antunes, Ana Paula Carvalho da Silva Ferreira, Lucas Freitas Berti, Ivan Moura Belo, Jamil Faissal Soni, Bertoldo Schneider

**Affiliations:** a NeuroMusculoskeletal System Unit (UNME), Hospital of the Federal University of Paraná (CHC UFPR), Curitiba, PR, 80060-900, Brazil; b Postgraduate Program in Electrical Engineering and Industrial Informatics (CPGEI), Federal Technological University of Paraná (UTFPR), Curitiba, PR, 80230-901, Brazil; c Academic Department of Mechanical Engineering (DAMEC) Federal Technological University of Paraná (UTFPR), Curitiba, PR, 81280-340, Brazil; d Graduate Program in Mechanical Engineering and Materials (PPGEM), Federal Technological University of Paraná (UTFPR), Curitiba, PR, 80230-901, Brazil; e Department of Mechanical Engineering (EMC) Federal University of Santa Catarina (UFSC), Florianópolis, SC, 88035-972, Brazil; f Faculty of Medicine, Pontifical Catholic University of Paraná, Curitiba, PR, 80215-901, Brazil

## Abstract

**Aims & objectives:**

Surgical treatment of Hoffa's fracture is often challenging for orthopedic surgeons. Current surgical techniques recommend stabilization of most of these fractures using a plate and screws. The objective this study was to design, simulate using Finite Element Analysis (FEA), and to describe the mechanical behavior of a specific orthopedic implant model to treat a Letenneur type III Hoffa fracture.

**Materials & methods:**

From CT scan images in DICOM format, a 3D bone model was created and modeled using Invesalius and Meshmixer software. The orthopedic implants were modeled by SolidWorks software. Subsequently, virtual surgical planning was performed with anatomical reduction of the fracture, including the selection and positioning of the implants. Ansys software was used to perform FEA of the fracture fixation types. Bone-implant systems were discretized, the mechanical and tribological properties were defined, boundary conditions were established, and force was applied based on literature data. Six domains (bone-implant systems) were defined according to the types and combinations of implants used in Hoffa fracture fixation. Mechanical stability was analyzed by measuring the relative displacement of bone fragments and the maximum von Mises stress in the implants.

**Results:**

FEA was assessed through visual analysis of the color gradient indicating bone displacement and implant stress. Quantitative measurements of bone displacement and maximum von Mises stress in the implants were performed. The FEA analysis showed that the system fixed with the developed implant model presented the smallest relative displacement of the distal bone fragment when subjected to a load of 1357.70 N.

**Conclusion:**

The developed implant model demonstrated the greatest mechanical stiffness, with the smallest relative displacement of the distal bone fragment when compared to traditional osteosynthesis methods for this type of fracture in the FEA.

## Highlights

• This study described the biomechanical analysis of a new orthopedic implant model.
• This study designed and simulated, a specific orthopedic implant model for the treatment of Hoffa fracture.
• The developed implant model presented great mechanical stiffness to fix the Hoffa fracture.

## 1 Introduction

Coronal fractures of the femoral condyle (Hoffa fracture) are associated with high-energy trauma, mainly related to urban violence, such as car and motorcycle accidents, pedestrian collisions, falls from heights, and physical aggression with firearms. These fractures are also related to osteoporosis in low-energy trauma, with an increase in the incidence of distal femur fractures, especially in the age group over 60 years in women.^1^ Coronal fractures of the femoral condyle account for between 0.65% and 1% of all femoral fractures and approximately 8% to 13% of fractures of the distal femur femoral fractures.^2^ According to some authors, approximately 3,000,000 femur fractures occur annually worldwide due to traffic accidents alone; therefore, approximately 19,500 cases of Hoffa fracture occur per year worldwide with this type of high-energy trauma.^3^ Due to the energy of the trauma and the geometry of the fracture, which are very unstable and difficult to treat surgically, often leading to postoperative complications such as osteonecrosis of the fragment, loss of reduction, nonunion, or malunion.^4^

To date, few surveys in the international medical literature have proposed biomechanical studies using computer simulations. Due to this scenario, it is interesting to carry out a study using numerical analysis with Finite Element Analysis (FEA), to investigate the mechanical characteristics of the force distribution that acts on the distal femur and the coronal fracture of the femoral condyle, and to estimate the kinds of osteosynthesis for the treatment of this type of fracture.

In recent years, in silico mathematical simulations in Computer-Aided Engineering (CAE) using FEA have become an excellent resource to increase the understanding of the biomechanics of the musculoskeletal system in physiological and pathological situations of the musculoskeletal system in physiological and pathological situations^5^(6). In the field of biomechanics, there are two main uses: analysis of body dynamics (kinetics of the musculoskeletal system) and analysis of stresses/deformations of systems (bone, tendons, joints, muscles, and implants).^6^ According to Taylor and Prendergast, historically, there are two reasons for performing FEA on orthopedic implants: to obtain a greater understanding of the behavior of the bone-implant system, and to develop a specific device, and to assist in the design and pre-clinical testing of new implants by comparing their performance with existing designs. With FEA simulations, it is possible to analyze the mechanical behavior of implants or prostheses, the biological responses of the bone to biomechanical changes after orthopedic surgeries, making it possible to prevent failures in fixing the implant to the bone, such as loosening or even fractures.^7^ A major challenge in carrying out a biomechanical analysis using FEA is to accurately reproduce the anatomy of the studied region and determine the perfect mechanical interaction between the structures.^7^ To achieve this objective, high-resolution medical images such as computed tomography (CT) have been used to create volumes of anatomical models that are rendered and modeled in an environment with Computer-Aided Design (CAD) technology, always taking care not to distort the original and natural geometry of the object. From then on, it is possible to perform different types of simulations, such as virtual surgeries, modeling of implants and prostheses (customized or not), and mathematical simulations to analyze mechanical behavior. Software with CAE technology is used as a tool so that volumes of virtual 3D anatomical models can be discretized and analyzed by the FEA. It is also possible to perform static and dynamic analyses, measuring the forces acting on the bone, the implant, and the bone-implant interface. In this way, it is possible to measure forces (compressive and traction) and deformation of the entire bone-implant system. This type of analysis is important in defining the best mechanical and geometric characteristics of an orthopedic implant (topological optimization), and it can even be customized or produced with additive manufacturing (AM) technologies - 3D Printing.^8^ CAE technology has made major technological advances in recent decades. Already in the 2010s, these advances related to biomechanical studies have high reproducibility and versatility, making it possible to achieve a high level of correlation between computational tests and conventional experimental mechanical tests of around 95%.^7^ An important advantage of studies using FEA is the obtaining of detailed data from the 3D model subjected to mechanical stress. During the computer simulation of the project, it is possible to measure the deformation and stress levels during different loading conditions in static or dynamic situations. This type of analysis helps to design and develop orthopedic implants with greater mechanical efficiency and a more anatomical design (design optimization) and with a lower potential risk of mechanical failure.^9^ FEA can be used to retrospectively evaluate and solve problems of complications or failures to avoid similar future occurrences. It is also an economic way to evaluate implants, procedures, and techniques in a shorter period compared to traditional mechanical testing methods with destruction of specimens^10^ .^11^

This study aims to describe the technological development of a specific orthopedic implant for the Hoffa fracture treatment. Furthermore, this study evaluated the mechanical stability of Hoffa fracture fixation (Letenneur type III), using a particular plate model for fixing this fracture compared to traditional fixation methods.

## 2 Materials & methods

### 2.1 Medical images acquisition

This study was carried out after approval by the Ethics Committee of the Technological University of Paraná – UTFPR, with registration under CAAE:57024722.6.0000.5547. The images in DICOM format from a CT scan of a patient presenting with Hoffa fracture (type III Letenneur) were used in this study. The data were acquired on a GE Lightspeed VCT (2008) tomograph with 64 channels, using a specific image acquisition protocol for bone tissue in the knee region, with a pitch of 512x512 and 0.6 mm slice thickness.

### 2.2 3D modeling (bone + implants)

Bone tissue segmentation was performed using the Invesalius software (v3.1.1, Renato Acher Information Technology Center – CTI, Campinas, Brazil). Bone segmentation was performed using an automatic software algorithm that identified bone tissue in the threshold window between 226 and 2014 HU (Hounsfield unit). The bone model was created using Meshmixer software (v3.5, Autodesk, São Rafael, USA).

The implants were modeled in a CAD environment using SolidWorks software (v.19.0, Dassault Systèmes S.A., France). The implants modeled were cortical and cancellous screws, linear and H-shaped plates. The plate design was shaped and adapted to the lateral surface of the distal femur to completely fit the bone surface.

The design of the H-shaped plate model (called H-shaped femoral condylar plate - HFCP) proposed in this study was developed according to mechanical principles to generate greater stability and rigidity: fracture fixation in two planes and fracture fixation with elements perpendicular to the fracture line by the principles of fracture fixation recommended by the AO foundation.^12^

Once the 3D objects were obtained, virtual surgical planning was carried out (bone model + implants) with fragment reduction, selection of the position and size of the implants. The fragments were virtually separated to better study the injury, and then reduced to the anatomical position, and the implants were positioned in the desired locations, which allowed greater mechanical stability of the fixation. The entire virtual surgery process was carried out using CAD software (Meshmixer and SolidWorks).

**Fig. 1:**
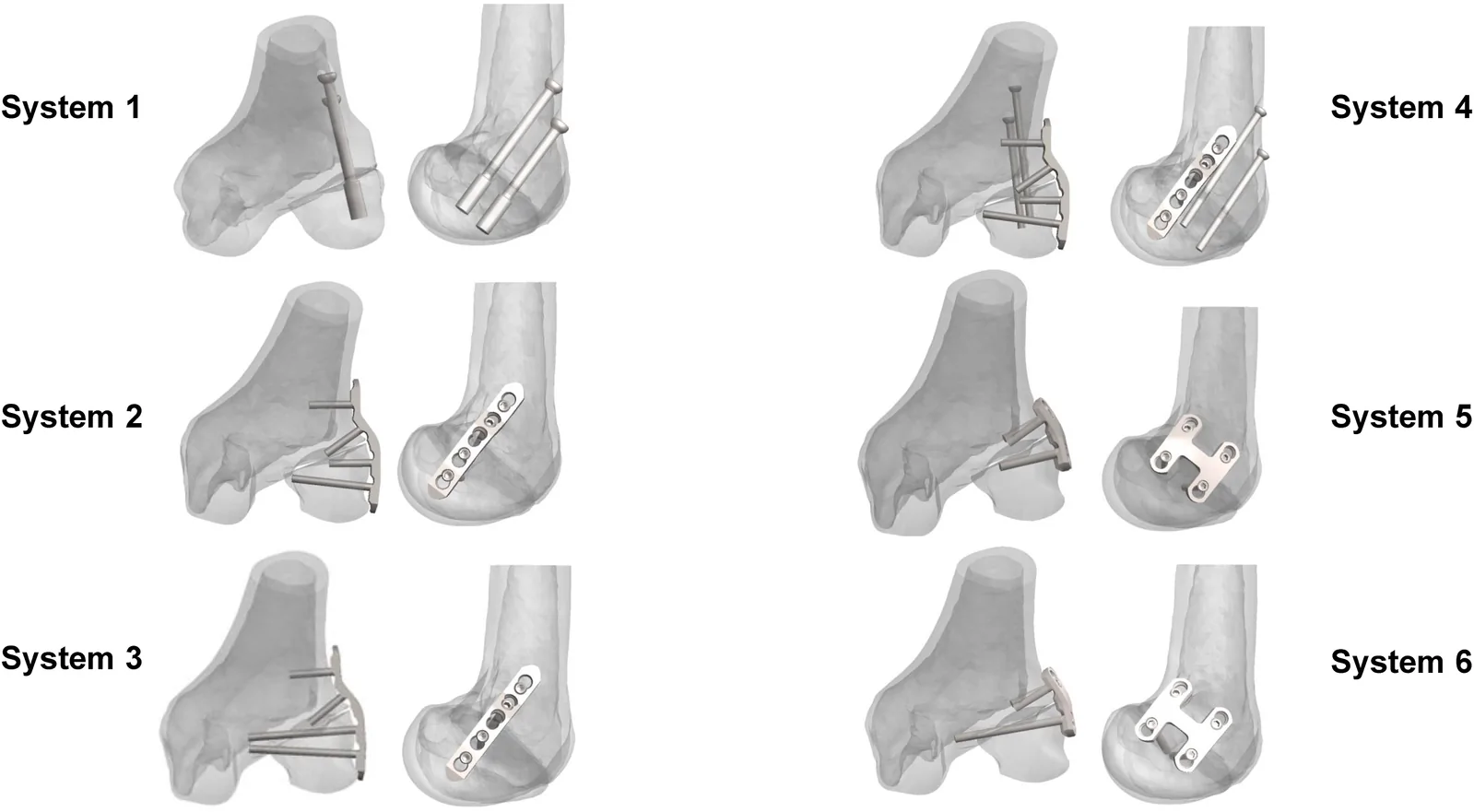
Bone-implant systems.

To perform the in silico biomechanical study, six bone-implant systems were defined as shown in Table 1 and Fig. 1. For the FEA, the screw geometry was simplified, making the entire screw body a uniform cylindrical structure — places where the thread contacts the bone (3.5 mm cortical screws and 3.5 mm locked screws). In 6.5 mm cancellous screws, the screw body had two diameters according to the thread area: the core of the screw has 4.5 mm in diameter and the thread area had a diameter of 6.5 mm, corresponding to the contact area with the bone. Various implant combinations were tested to define mechanical behavior (stiffness assessment) of the bone-implant systems.

**Table 1:** Total number of elements and nodes from each domain (bone-implant system).

Bone-Implant System		Total Number	
System	Fixation Type	Elements	Nodes

1	2 cancellous screws 6.5 mm with partial thread 16 mm anterograde	443.188	661.707
2	5-hole linear locking plate with 4 × 3.5 mm locking screws (2 distal screws)	440.847	659.765
3	5-hole linear locking plate with 4 × 3.5 mm locking screws (2 distal screws fixing distal femur/interfragmentary screws)	445.077	667.032
4	5-hole linear locking plate with 4 × 3.5 mm locking screws (2 distal screws) + 2 × 4 mm antegrade parallel cancellous screws	464.475	696.852
5	CFP with 4 locking screws 3.5 mm (2 distal screws)	459.429	687.656
6	CFP with 4 locking screws 3.5 mm (2 distal screws fixing the distal femur/interfragmentary screws)	461.045	690.609

### 2.3 Meshing and material properties

To perform the numerical simulation (FEA), it was determined the domain's geometry (objects to be studied, in this case, the 3D bone model + implants), which were discretized into a mesh of connected elements. Manual mesh convergence was carried out, and the discretization of the most “critical” points of the bone-implant system was increased. In the mesh convergence study, the size of the elements was evaluated as a function of their displacement using the bone-implant system 4 as the domain. Convergence occurred after 1 mm mesh element size. The elements of the anatomical model and implants were defined as 10-node tetrahedral elements, following current literature, due to greater simulation accuracy.

In the present study, based on recent studies on FEA in orthopedics [13-18] all materials were assumed to be isotropic and homogeneous with linear elastic behavior. The mechanical and tribological properties are summarized in Table 2, Table 3. This study is part of a research into the development of additive manufacturing of orthopedic implants. The implant developed in this study was manufactured using AM technology, specifically Electron Beam Melting (EBM) with Ti_6_Al_4_V alloy on equipment from the Arcam company. The screws were manufactured using a subtractive manufacturing technique from grade 23 Ti_6_Al_4_V bars of 6 and 8 mm with the following mechanical and tribological properties according to the supplier (Table 2).

**Table 2:** Mechanical properties of materials related to orthopedic implants.

Properties	Cortical Bone	Ti_6_Al_4_V Arcam®	Ti_6_Al_4_V Grade 23
Modulus of Elasticity (MPa)	17.900	120.000	113.800
Poisson's coefficient	0.3	0.34	0,34
Compressive yield stress (MPa)	182	930	805
Tensile yield stress (MPa)	115	950	820
Ultimate tensile strength in compression (MPa)	195		
Ultimate tensile strength (MPa)	133	1020	1093

**Table 3:** Static friction coefficient of materials related to orthopedic implants.

	Friction coefficient
Cortical bone – Cortical bone	0,46
Cortical bone - Titanium alloy (Ti_6_Al_4_V)	0,3

### 2.4 Loads and boundary conditions

The contacts between the screws and the plate and between the screws and the bone were established as bonded contacts, while the contacts between the bone fragments and between the plate and the bone were defined as frictional, according to the Ansys software (v22.0, Canonsburg, USA). The region of the cancellous screws that have bonded contact with the bone was only in the region of the fracture distal fragment. This was defined to generate the compression effect (preload boundary condition). In the proximal region of the cancellous screws, no type of contact was considered.

In this study, ligamentous and/or muscular actions, as well as the articular contact of the femur surface with the tibia on the bone-implant system, were not considered. Also, the loading occurred as an instantaneous event over 1 s, according to the automatic algorithm of the Ansys software.

To carry out this research, the force application area on the articular surface of the fracture distal fragment in the lateral femoral condyle of the knee was determined. This contact area corresponds to the contact of the lateral femoral condyle on the knee with 10° flexion. This degree of flexion was defined according to a biomechanical study by Protopapadaki et al., which details aspects of kinematics and kinetics of gait during the ascent and descent of stairs in healthy young individuals. The author describes that the load peak due to the vertical reaction force of the ground on the knee during stair descent occurs at 10% of the descent cycle time. At this moment, the knee is flexed at around 10°.^13^

We assumed that the load condition in this study was the most critical physiological load acting on the knee during everyday activities, according to Kutzner. This load condition represents the greatest physiological load supported by the knee when descending stairs during daily activities, corresponding to 346% of body weight.^14^

In the structural simulation of Hoffa fracture (Letenneur type III) fixation, we assumed the same distribution of loads between the medial and lateral condyles as proposed by Huang, Xiaowei et al., and Chen, Pengbo et al.: 60% of compressive loading on the medial condyle and 40% on the lateral condyle. We also considered an individual with a mass of 100 kg and a gravity force of 9.81 m/s^2^ (981 N representing body weight - BW). Considering the effort acting on the knee when descending stairs, the force corresponded to 1357.70 N under the lateral condyle^15^ .^16^

To evaluate the osteosynthesis rigidity with the HCFP in an immediate postoperative situation, a simulation of partial support of the operated limb of a 100 kg person was carried out considering data presented by Eickhoff et al., with partial support of the operated limb weighing 35 kg, representing a force applied to the lateral condyle of 137.34 N.^17^

### 2.5 Biomechanical analysis

The interfragmentary displacement of the fracture was evaluated using system 6 to assess the interfragmentary strain. Visual analysis (quasi-quantitative) was performed according to the color gradient presented in the software legend, allowing the regions of greatest displacement and/or stress to be quickly visualized. By visualizing the domain (bone-implant system), it is possible to identify areas of bone displacement and areas of stress concentration in the implants. The region of the distal diaphysis of the femur was restricted in the degree of translational freedom. To carry out this study, the effect of gravity was considered negligible in the bone-implant system.^18^ In our study, pre-loading was used on the cancellous screws corresponding to a compressive load of 2000 N. In our study, pre-loading was used on the cancellous screws corresponding to a compressive load of 2000 N. This arrangement promotes compression of the bone fragments and respects the result of stresses generated in the screws being lower than 85% of the test load of the material considered.

**Fig. 2:**
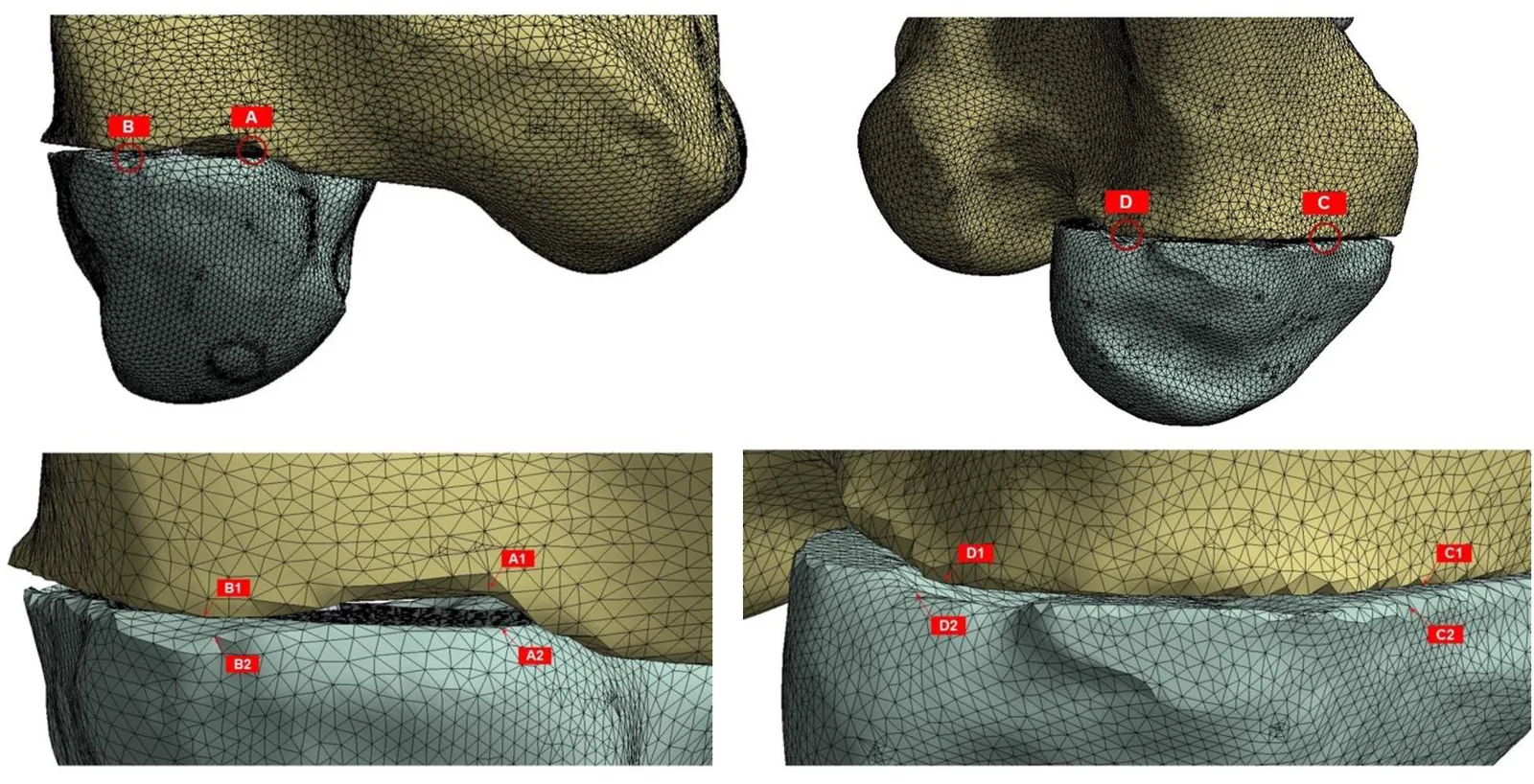
Points for analysis of fracture fragment displacement. Note: A and B points (anterior view). C and D points (posterolateral view).

In the quantitative evaluation of the data obtained, values of absolute (mm) and relative (%) displacements between the fracture fragments were analyzed to determine the interfragmentary displacement. This was done by defining points of interest at the contact interface between the fragments based on the anatomical importance of the locations, such as the articular surface. Four points were defined to determine the positioning at the interface between the fragments (fracture line). There were two anterior points (A and B), one lateral point (C), and one posterior point (D) according to the researcher's previous analysis to define the contact critical points between the fragments (Fig. 2). The two articular points were critical locations on the articular surface of the lateral femoral condyle. Similarly, the von Mises stress variations and the maximum von Mises stress peaks of the bone-implant system were discriminated and highlighted to analyze the system's stiffness. To carry out the quantitative analysis of absolute displacements, linear displacements in millimeters (mm) were defined. In this way, it was possible to determine relative positions and interfragmentary positioning. This allowed analysis of the fracture consolidation potential according to the interfragmentary strain rate. In this study, the interfragmentary strain was determined according to Perren's research, as shown in Equation (1):

(1) *E = (DL / L)* ∙ 100 %

were:

*E* = Interfragmentary strain
*DL* = final crack size - initial crack size
*L* = initial crack size

For this purpose, the points to be studied immediately opposite the fracture focus were defined, and distances were measured in mm in the X, Y, and Z coordinates, with the Z axis corresponding to the longitudinal axis of the bone. After applying the force, the resulting linear displacement was defined. From these data, the relative displacements of each point were calculated according to the equation.

## 3 Results

### 3.1 Visual analysis (quasi-quantitative)

**Fig. 3:**
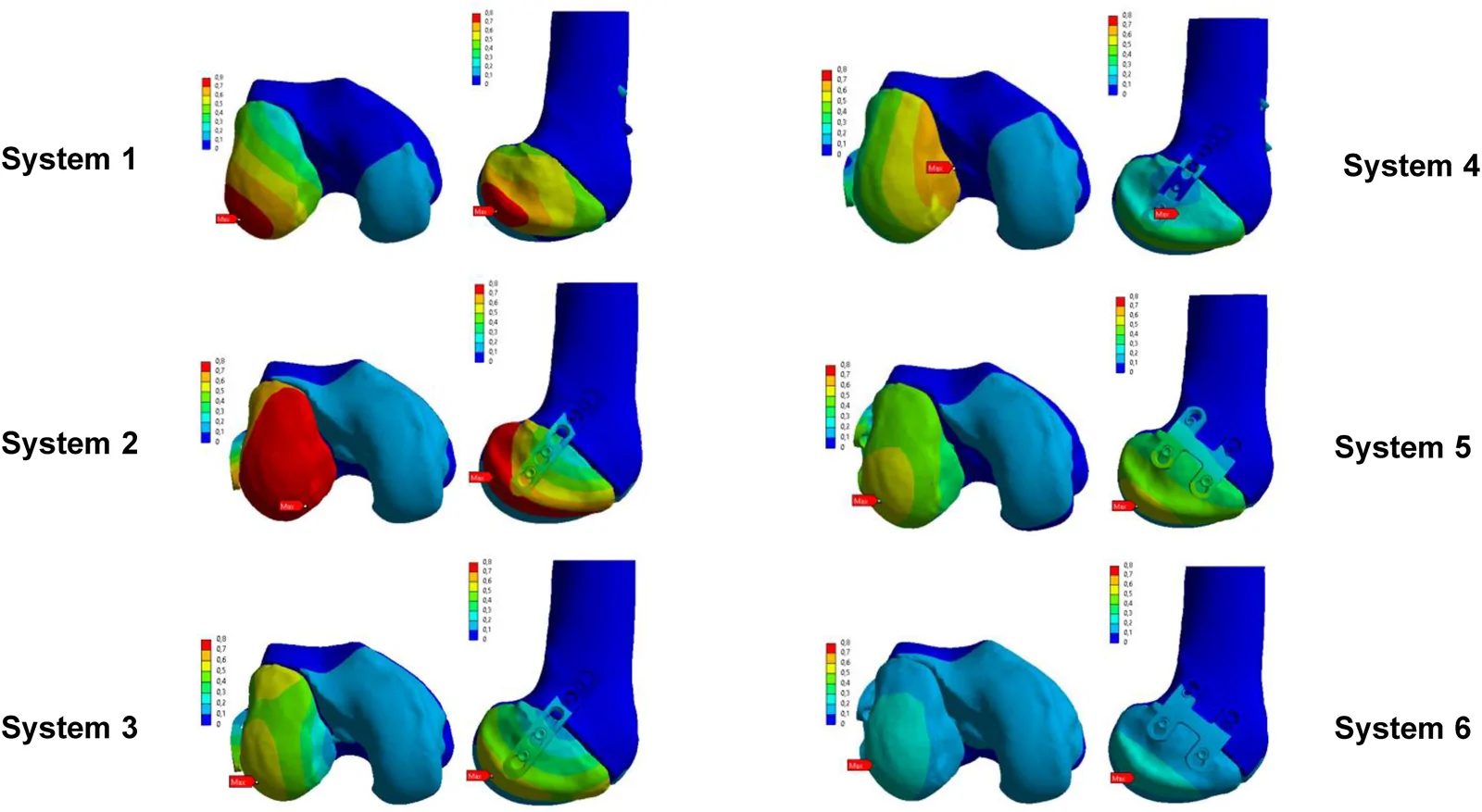
Maximum displacement of the distal fragment (mm) in systems 1 to 6.

**Fig. 4:**
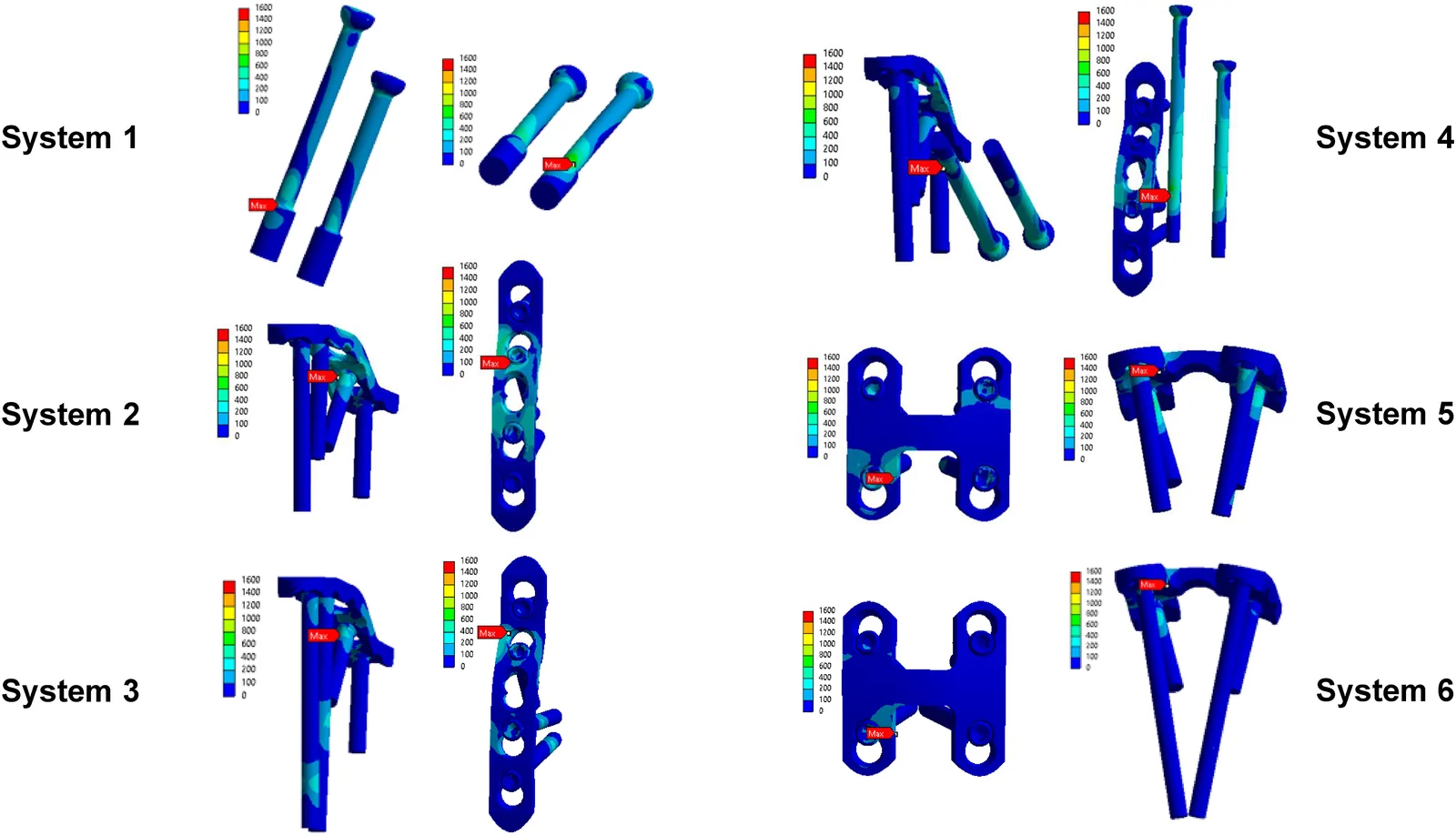
Maximum von Mises stress (MPa) in systems 1 to 6.

During visual analysis, it was observed that there were points of maximum displacement (Fig. 3) and points of maximum von Mises stress concentration in the implants (Fig. 4). The areas of stress concentration were very small and did not compromise critical areas of the implant structure.

### 3.2 Quantitative analysis

Quantitative analysis was performed by measuring the maximum displacement of the distal fragment and the displacement of the defined points (four points – A, B, C, and D) (Fig. 2) to determine the relative displacements (interfragmentary strain) according to Equation (1).

**Fig. 5:**
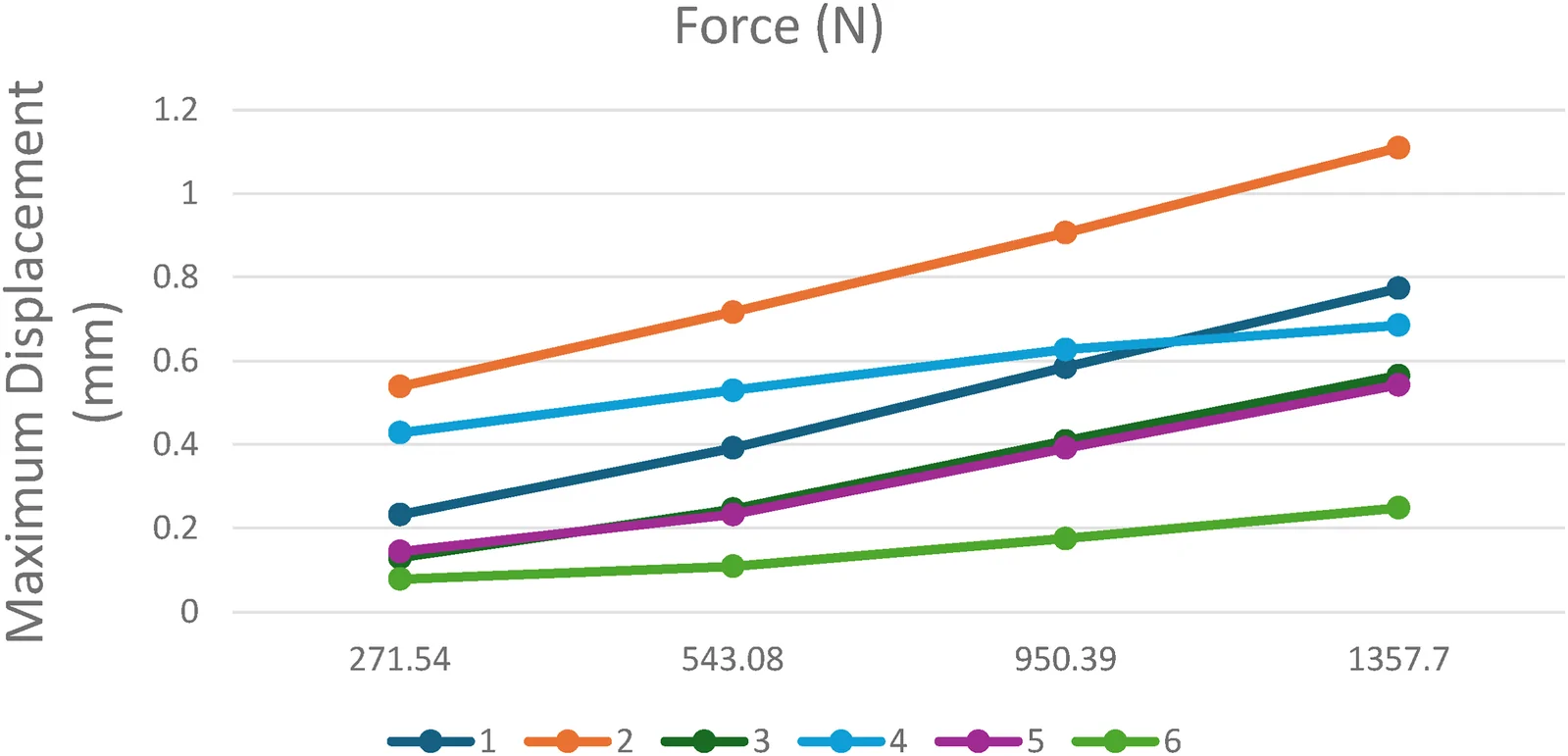
Maximum displacement of the distal fragment (mm) as a function of the force (N) applied to the system.

**Fig. 6:**
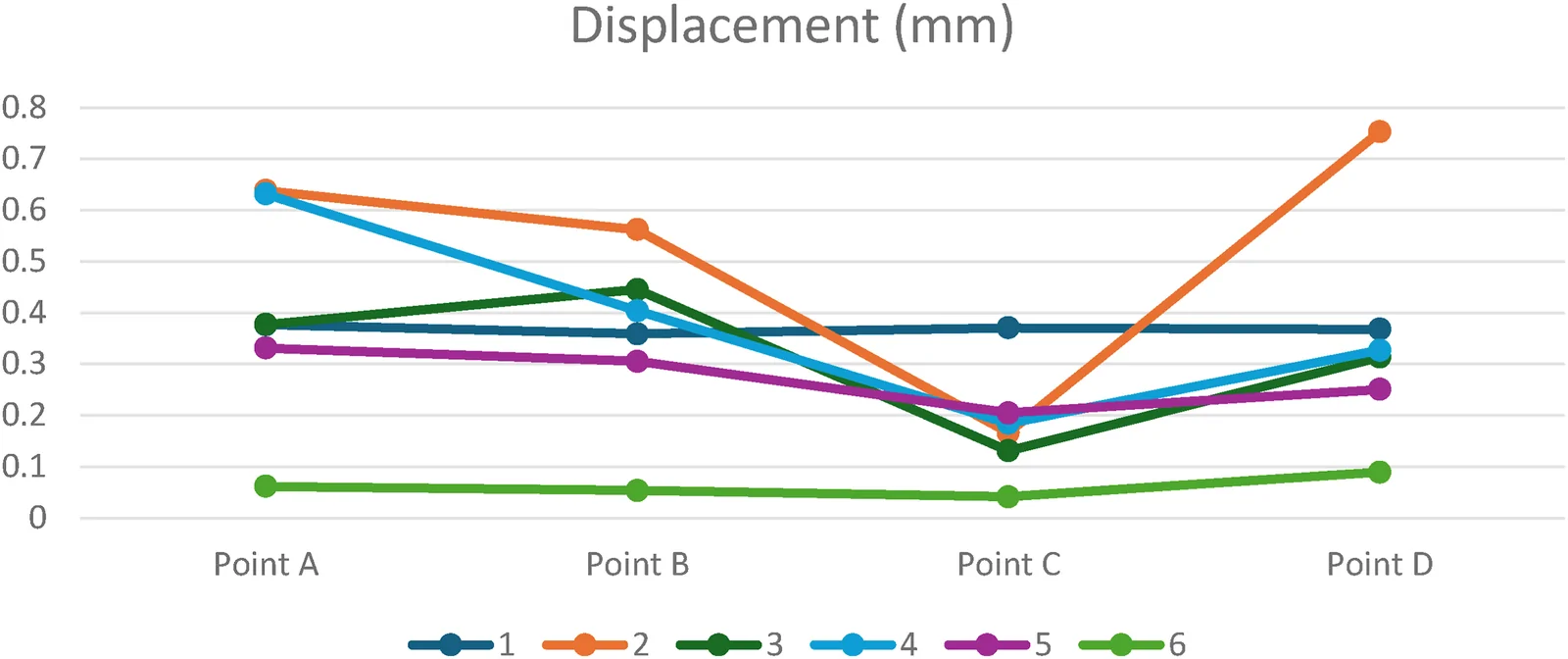
Displacement (mm) as a function of the system points.

**Fig. 7:**
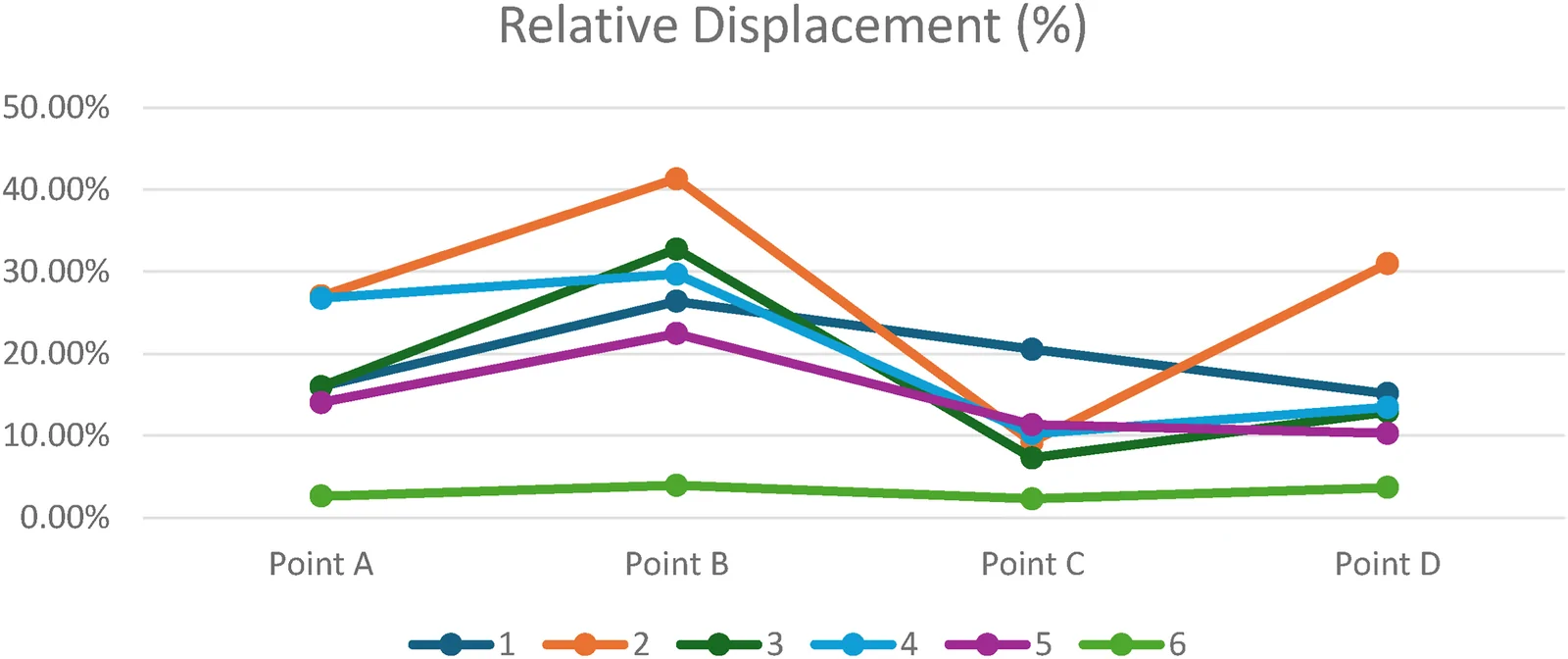
Relative displacement (%) as a function of the system points.

**Fig. 8:**
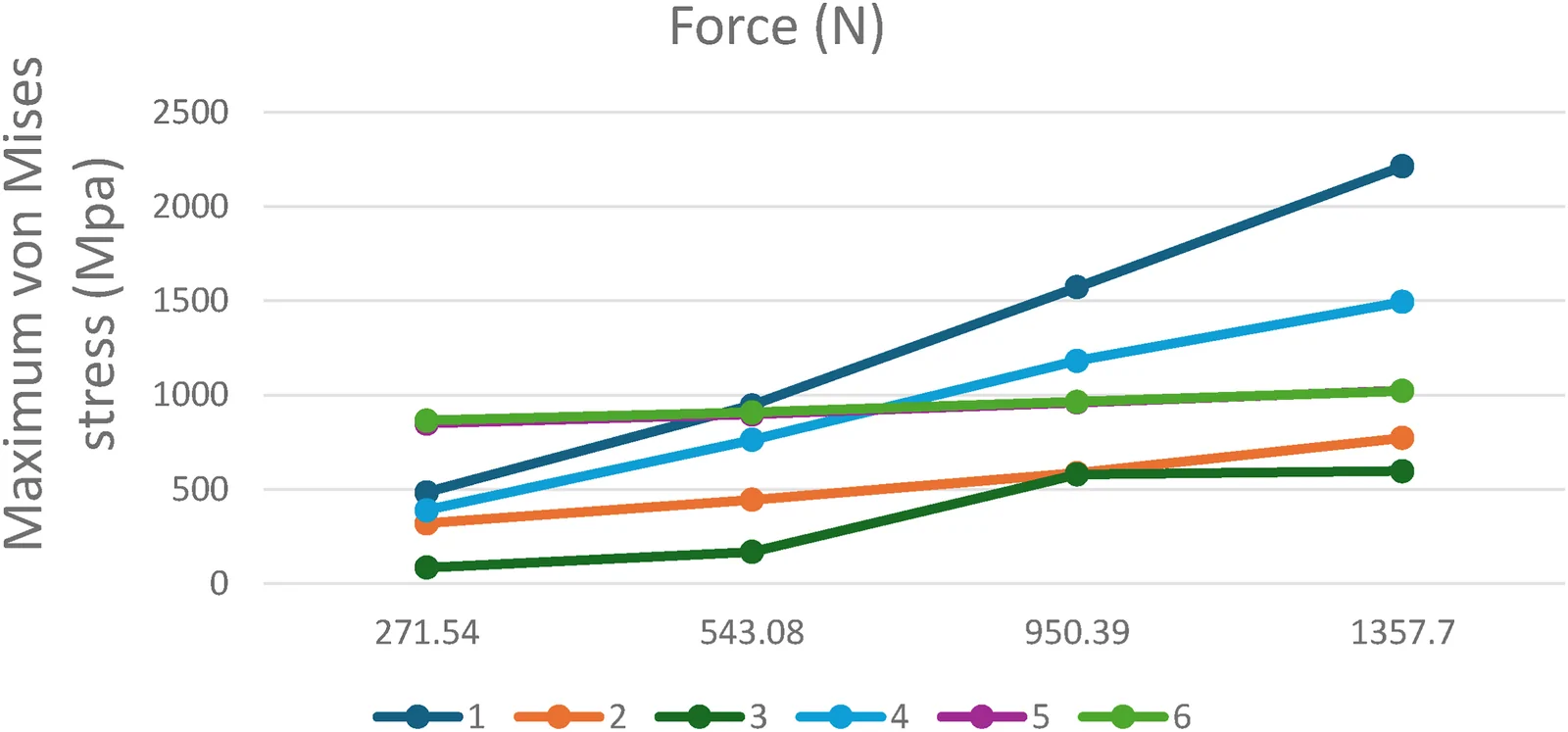
Maximum von Mises stress (MPa) as a function of the force (N) applied to each system. Note: The lines of system 5 and 6 are overlapping.

According to Fig. 5, the variation in the maximum displacement of the distal fragment (mm) as a function of the force (N) applied is directly proportional in all systems. The values of absolute displacement and relative (interfragmentary) displacement at each point can be seen in Fig. 6, Fig. 7, and Tabela 4, Table 5. According to Fig. 8, the variation in maximum von Mises stress (MPa) as a function of the force (N) applied is directly proportional. The local concentration of implant stress (maximum von Mises stress) can be seen in Table 6.

**Tabela 4:** Displacement (mm) and Interfragmentary Strain (%) in each system.

System	Maximum Displacement (mm)	Medium Displacement (mm)	Maximum Interfragmentary strain (%)	Minimum Interfragmentary strain (%)	Medium Interfragmentary strain (%)
1	0,38	0,37	26,38%	15,11%	19,49%
2	0,75	0,53	41,31%	9,20%	27,12%
3	0,44	0,31	32,73%	7,26%	17,20%
4	0,63	0,39	29,75%	10,22%	20,02%
5	0,33	0,20	22,44%	10,27%	14,51%
6	0,09	0,04	3,92%	2,27%	3,11%

**Table 5:** Maximum and minimum interfragmentary strain (%) in each system.

System	Maximum Interfragmentary strain (%)	Point	Minimum Interfragmentary strain (%)	Point
1	26,38%	B	15,11%	D
2	41,31%	B	9,20%	C
3	32,73%	B	7,26%	C
4	29,69%	B	10,22%	C
5	22,44%	B	10,27%	D
6	3,92%	B	2,27%	C

**Table 6:** Minimum and maximum de von Mises stress (MPa) when applying a load of 1357,70 N to each system.

System	Minimum von Mises stress (MPa)	Maximum von Mises stress (MPa)	Location of the maximum load peak von Mises stress in the implant
1	7,87E-03	2.214,40	Proximal anterograde cancellous screw: The interface region between the fragments
2	9,799E-07	772,95	Linear lock plate: Region of the proximal locked screw hole to the side of the fracture line
3	2,495E-06	596,87	Distal locked screw near the fracture line: Region of the interface between the fragments and the region on the roof of the intercondyle
4	1,250E-05	1.495,30	Proximal anterograde cortical screw: Interface region between the fragments
5	4,432E-03	1.204,10	Region of the distal and posterior locked screw hole of the CFP
6	9,842E-03	1023,20	Region of the distal and posterior locked screw hole of the CFP

**Fig. 9:**
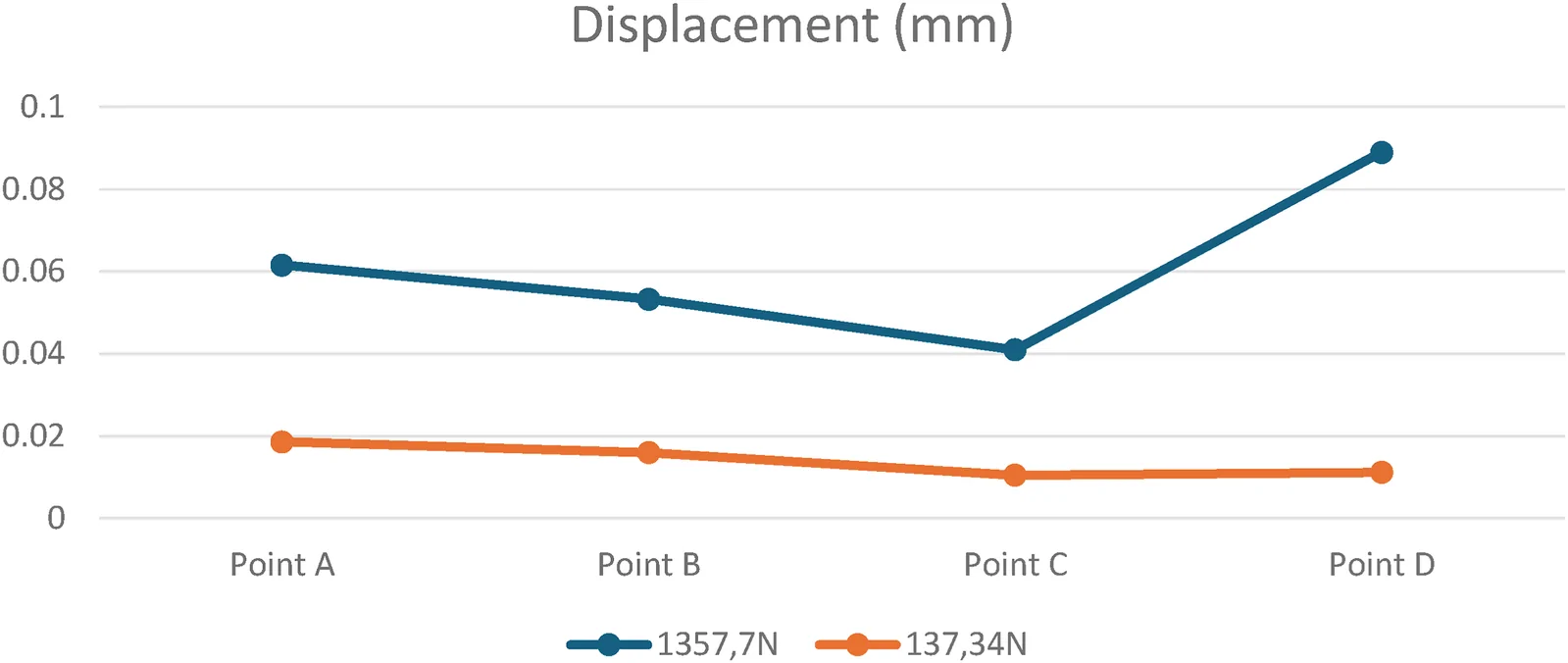
Interfragmentary strain in the system 6 when forces of 137,34 N and 1.357,70 N are applied.

**Fig. 10:**
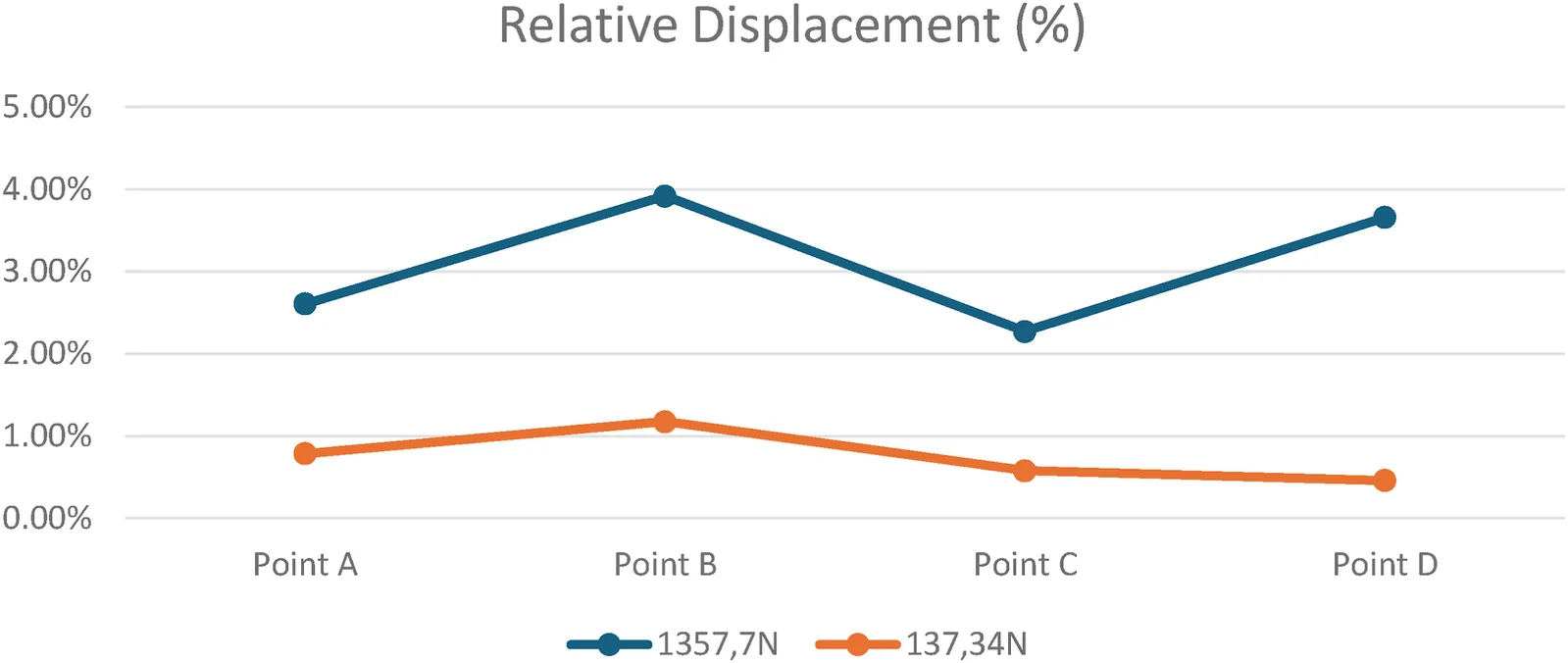
Interfragmentary strain in the system 6 when forces of 137,34 N and 1.357,70 N are applied.

A simulation was performed to study the interfragmentary strain of CFP (using system 6) in an immediate postoperative situation, simulating partial support of the operated limb using two crutches (35 kg representing a force applied to the lateral condyle of 137.34 N) of a 100 kg person. It was noted that there was very small absolute and relative displacement, favoring adequate bone consolidation of the joint region (interfragmentary strain <2%) (Table 7, Table 8) (Fig. 9, Fig. 10).

**Table 7:** Maximum/minimum displacement and maximum/minimum interfragmentary strain in system 6 when a force of 137,34 N is applied.

System	Maximum Displacement (mm)	Medium Displacement (mm)	Maximum Interfragmentary strain (%)	Minimum Interfragmentary strain (%)
6	0,018	0,014	1,17	0,46

**Table 8:** Maximum and minimum interfragmentary strain at the fracture line points in system 6 when a force of 137,34 N is applied.

System	Maximum Interfragmentary strain		Minimum Interfragmentary strain		Medium interfragmentary strain
	(%)	Point	(%)	Point	(%)
6	1,17	B	0,46	D	0,75

## 4 Discussion

Due to the anatomical characteristics of the Hoffa fracture and the forces acting on the knee region, adequate fixation often becomes a challenge for the orthopedic surgeon. In many cases, fixation methods using screws alone do not provide the necessary mechanical stability to occur an adequate bone consolidation. Until the 2010s, most studies in the literature indicated fixation with screws only. Approximately 15 years ago, scientific studies and techniques appeared reporting the use of plates and screws in Hoffa fracture treatments, which showed greater mechanical stability in this type of fracture fixation. Therefore, few articles in the literature present studies on the use of plates and screws in Hoffa fracture treatments. According to Sun, the use of screws alone may be inadequate for the treatment of Hoffa's fracture and the use of a plate may be necessary, with possible positions being lateral and posterior. The posterior plate can be considered a buttress, and the lateral plate as neutralizing shear forces following the principles of AO.^19^ In recent years, there have been case reports of the use of plates and screws in the treatment of Hoffa fractures^20^ ^21^ ^22^ ^23^ ^24^ ^25^ ^26^ ^27^

In our previously published study, we described the application of 3D printing technology in the treatment of Hoffa fracture pseudarthrosis, describing a clinical case of Hoffa fracture that underwent surgical treatment with two anterior traction screws and a neutralization locking plate in the lateral condyle. In this case, there was great accuracy in the reproducibility of virtual planning and surgical simulation with the 3D printed anatomical model in the surgical treatment of Hoffa fracture nonunion.^28^

Sun et al., in a biomechanical study, concluded that positioning the lateral plate is the most stable method of fixing Hoffa fractures type Letenneur I, and that the use of the plate is not indicated for all cases. This author carried out a study comparing four groups: group one with a 3.5 mm posterior locked plate and a 6.5 mm cannulated screw in a posteroanterior direction, group two with two parallel cannulated screws 6.5 mm in a posteroanterior direction, group three with lateral 3.5 mm locked plate and a 6.5 mm cannulated screw in a posteroanterior direction, group four with two parallel 6.5 mm screws in an anteroposterior direction. The two groups with plates demonstrated greater mechanical resistance (axial rigidity) than the groups with screws alone. The group with the lateral plate showed greater mechanical stability than the other three groups.^19^

Pires et al. proposed an algorithm for treating lateral Hoffa fractures according to Letenneur's classification: Letenneur I: posterolateral approach with fixation with an anti-slip plate and screws from anterior to posterior; Letenneur IIa, IIb, IIc: posterolateral access with screw fixation in the posterior to anterior direction; Letenneur III: lateral parapatellar access using screws from anterior to posterior associated with a neutralization plate.^29^ The same author also proposed an algorithm for the treatment of fractures of the medial condyle: posteromedial access with fixation with a posteromedial anti-slip plate associated with screws depending on the type of fracture.^30^ In a recent biomechanical study comparing four different types of internal osteosynthesis for Hoffa fracture Letenneur type I, Pires et al. concluded that the use of the posterolateral reinforcement plate associated with screws inserted in the posteroanterior direction continues to be the biomechanical gold standard in the treatment of type I Letenneur fractures. The use of isolated screws inserted in the anteroposterior direction should be avoided in fractures of Letenneur type I due to its poor biomechanical performance. The anteroposterior screws associated with the locked third-tube plate placed horizontally perpendicular to the main fracture plane increased the fixation rigidity by 302% compared to the isolated anteroposterior screw fixation.^31^

Peez et al. conducted a biomechanical stuy simulating comminuted lateral Hoffa fracture (Letenneur type II) in human cadaveric distal femur. The fractures were fixed after anatomic fracture reduction with either isolated crossed posteroanterior screws (PA screws – two parallel 4.5 mm fully-threaded cortical screw) or additionally with either a posterior plate (PA screws + posterior plate - 3.5 mm 6-hole locking compression plate), a lateral locking plate (PA screws + lateral plate - 3.5 mm 3-hole PHILOS plate), or combined posterior and lateral locking plates (PA screws + double plate). All specimens were biomechanically tested. This author concluded that plate-augmented posteroanterior screw fixation of comminuted Letenneur type IIb Hoffa fractures provided greater biomechanical stability than isolated posteroanterior screw fixation. While additional lateral or double plate fixation improves the stability of both the intercalary and Hoffa fragment, posterior plating stabilized only the Hoffa fragment.^32^

In the literature, there is an increasing, but still small number of studies developed on mechanical analysis by FEA to evaluate the behavior of fractures and orthopedic implants. Regarding the use of FEA to evaluate the behavior of the Hoffa fracture, the study by Freitas et al. analyzed four fixation methods for Letenneur type II Hoffa fractures: two cannulated screws of 7 mm from anterior to posterior (anterograde), two cannulated screws of 7 mm from posterior to anterior (retrograde), two cortical screws of 4.5 mm from anterior to posterior and two cortical screws of 4.5 mm from posterior to anterior. It was evaluated the total displacement and maximum von Mises stress in the implants. They concluded that the fixation system with a 7 mm cannulated screw presented the best mechanical results evaluated by FEA in the treatment of Letenneur type II Hoffa fractures, causing a decrease in vertical displacement when used in retrograde and in peak von Mises stress in anterograde.^33^ Jia, Chang, and Tang (2025) performed an FEA to simulate the biomechanical differences between anterior-posterior (AP) direction and posterior-anterior (PA) direction placement of two cannulated screws in Hoffa fractures. The author simulated Letenneur IIa, IIb, IIc, III Hoffa fractures, and two groups of screw internal fixation models were constructed. Two 6.5 mm cannulated screws were implanted parallel in the AP direction or the PA direction. The biomechanical test was performed to determine the displacement, stress distribution, and peaks in the distal femur and cannulated screws. This author concluded that the mechanical stability of the two screw insertion methods is similar. Inserting screws from anterior to posterior can reduce the stress on the distal femur and minimize the dissection of the posterior soft tissues.^34^

Most articles that performed biomechanical studies using FEA for osteosynthesis analysis only analyzed the stress distributions of the implants and not the bone displacement. Both, the fragment bone displacement and even more so, the fracture relative displacement (interfragmentary strain), are the greatest predictors of adequate bone healing, as shown in studies by Perren^35^ ^36^.^37^ According to this author, in his experimental biomechanical study on the phases of bone healing, observed that for the fracture to heal, relative interfragmentary displacement (interfragmentary strain) must be less than 10%. An interfragmentary displacement greater than 10% does not form a bone callus, only cartilage and/or fibrous tissue.^36^ In joint regions, due to mechanical demands, osteosynthesis must be as rigid as possible without mobility in the fracture focus, bone fragment reduction must be anatomical, allowing direct healing without the formation of bone callus and complete restoration of the articular cartilage. Therefore, in direct healing, the interfragmentary strain for this type of healing must be up to 2%^35^ .^36^ According to Fan et al., the two main mechanical requirements of an orthopedic implant for fracture treatment are to promote fracture stability to allow bone healing and to have mechanical rigidity that prevents the orthopedic implant from breaking. Bone healing occurs when the relative displacement between the fragments is less than 10%. The maximum von Mises stress values in the locked plate must be lower than the maximum yield stresses of the implant material so that the implant does not fail, and the fracture heals.^38^

Regarding the displacement of the bone fragment, the maximum displacement (mm) was observed in System 2 in the region of the internal face of the lateral femoral condyle in the posterior intercondylar region (1.10 mm) and the minimum displacement was observed in system 6 (0.25 mm) in the region of the lateral face of the lateral femoral condyle. It is also possible to verify whether there was attenuation of the change in stress or displacement, or whether there were abrupt changes in stresses and displacements in a region of the domain. In System 2, there was displacement of the distal fragment, generating an abrupt change in the color gradient of a large part of the distal fragment. In system 6, there was a smooth change in the color gradient, indicating a progressive distribution of the displacement.

According to Fig. 5, the variation in displacement (mm) as a function of the applied force (N) is directly proportional in all systems, which corroborates the expected behavior of a domain with elastic properties following Hooke's Law. Systems 3, 5, and 6 show a small change in the slope of the straight line when applying a force of 543.08 N. This can be explained by the interactions of forces between the bone fragments and the implants. Systems 2 and 4 presented the greatest variation in maximum displacement (mm) at the 4 points evaluated (A, B, C, and D). System 6 presented the smallest variation in maximum displacement (mm) at the 4 points evaluated. It was also observed that system 2 had the greatest variation in interfragmentary strain. The smallest variation in interfragmentary strain was observed in system 6. According to the data found on interfragmentary strain at the different defined points, it can be noted that the relative displacements at different points in the same system do not vary in the same proportion (they are not directly proportional). The place with the highest interfragmentary strain in all systems was point B, located on the articular surface, and those with the lowest interfragmentary strain are at the lateral and posterior points (Table 5). Systems 5 and 6 have the lowest maximum and minimum interfragmentary strain at points that were not located on the articular surface. Systems 5 and 6 had an average interfragmentary strain of 14.51% and 3.11%, respectively, when subjected to a force of 1357.70 N, simulating a 100 kg person descending a stair with full load on the studied limb. Considering an ideal situation of partial loading of the operated limb of a 100 kg patient, with immediate support of 35 kg, this represents a force applied to the lateral condyle of 137.34 N. When we compared the fixation of a Hoffa fracture (Letenneur type III) using a HCFP to fixation with only two traction screws, the latter presented an interfragmentary strain (19.49% vs 3.11%) 6.26 times lower (Table 4). According to the data found in the FEA, it was observed that the systems with the best biomechanical behavior to achieve adequate bone healing of the Hoffa fracture were systems 5 and 6. These systems present the lowest interfragmentary strain when subjected to the tested load that corresponds to a critical postoperative situation. The HCFP design provides fixation in two planes in addition to allowing interfragmentary fixation through the plate when using long distal screws that fix the intercondylar roof region, a region of dense cortical bone. According to the analysis of the displacement of the distal fragment and the displacement measured at the four points evaluated, systems 5 and 6 present the lowest averages of the smallest displacements. This shows the stiffness of these systems when applied to a load of 1357.70 N. When subjected to a load of 137.34 N, which corresponds to a usual postoperative situation with partial support of the operated limb, the interfragmentary strain observed in osteosynthesis with HCFP and distal screws fixing the intercondylar region is highly favorable to bone healing of the articular surface as indicated in the literature.

To analyze the distribution of maximum von Mises yield stress by FEA, the stress distribution in the implants was examined when applying the maximum load of 1357.70 N. During qualitative analysis, it was observed that there were points of maximum von Mises stress concentration (MPa) in the implants in very small areas, not compromising extensive areas. Maximum stress was observed in system 1 in the proximal screw (the cylindrical region of the screw corresponding to the fracture region between the fragments) (2214.40 MPa). Minimum stress was observed in system 2 (9.799E-07 MPa). According to Fig. 9, the variation of the maximum von Mises stress (MPa) as a function of the force (N) applied is directly proportional, except for System 3.

Similarly, regarding fragment displacement relative to the femur, the analysis was performed when applying maximum force of 1357.70 N. System 2 showed the largest maximum displacement (mm) and the smallest minimum von Mises stress (MPa). The highest maximum von Mises stress (MPa) was observed in System 1, with the second smallest maximum displacement. This indicated that the more rigid the system behaved, the greater the stress was distributed on the implants to keep the fragments together. System 6 presented the smallest maximum displacement (mm) and system 3 the highest maximum von Mises stress (MPa).

During visual analysis, it was observed points of maximum von Mises stress concentration (MPa) in the implants with very small areas that did not compromise extensive areas of the implant. Quantitative analysis also showed that the maximum von Mises stress was below the maximum yield stress value for the material (Ti_6_Al_4_V) of 1020 MPa in systems 2, 3, 5, and 6, indicating no possibility of plastic deformation with catastrophic implant failure during the application of the load of 1357.70 N.

The quantitative analysis showed that only in system 1 was there a maximum yield stress value for Ti_6_Al_4_V above 2000 MPa in a small area of the screws. In systems 5 and 6, the maximum yield stress value for Ti_6_Al4V was in the region close to the distal hole of the screw of the locked plate, indicating no possibility of plastic deformation with catastrophic implant failure during the application of the load of 1357.70 N. Only Systems 1 and 4 presented values higher than the maximum von Mises yield stress for Ti_6_Al_4_V (Fig. 8), showing a tendency towards plastic deformation if the force is maintained and there is no bone deformation, which could lead to catastrophic failure in the system. Systems 2 and 3 presented the lowest average stresses in the implants. Systems 5 and 6 presented the smallest differences in von Mises stress in the implants.

The isolated analysis of stress distribution only shows signs that the implant may present elastic or plastic deformation with possible catastrophic failure. Biomechanical studies that analyze implant behavior show that long before catastrophic failure occurs, there is bone deformation with fixation reduction loss. From a mechanical point of view, the analysis of the stress distribution is very useful in the dynamic mechanical analysis of implants after cyclic application of mechanical stress over a long period, with the possibility of fatigue. In static tests that evaluate implant fixation rigidity in the immediate postoperative period without considering boundary conditions such as bone healing, interfragmentary strain is considered a more reliable and accurate indicator of the possibility of adequate bone consolidation. As seen previously, systems that presented high proportional concentrations of maximum von Mises stress presented low relative displacements (Systems 1 and 4), and the opposite was documented in systems with low stress concentrations in the implants but high relative displacements (System 2). We consider that only analyzing the distribution of maximum von Mises stress in the implants may be a methodological failure in mechanical analysis by FEA that analyzes fracture fixation because it does not analyze the potential healing bone of these fixations (relative displacements).

The present study showed the following limitations: muscle-ligament interactions were not considered as elements of dynamic stability. Also, in a such controlled study environment, variations in the mechanism of injury were not tested, neither were fixations tested for all types of the Letenneur classification. Also, simplifications of screw fixation to the bone (screw thread) were assumed. Further studies are necessary to better understand this rare type of injury, bringing improvements in fixation techniques and a reduction in the rate of complications related to its treatment.

## 5 Conclusion

The present study developed an orthopedic implant design adapted to the lateral aspect of the lateral femoral condyle, specifically for the treatment of Letenneur type III Hoffa fracture. The FEA of the Hoffa fracture fixation systems, simulating a critical situation in daily life (stair descent) and a conventional postoperative situation, showed that the developed implant (HCFP) presents greater mechanical stability compared to the other tested implants for the treatment of Letenneur type III Hoffa fracture.

## Ethical approval and patient consent

This study was conducted after approval by the Ethics Committee of the Technological University of Paraná – UTFPR, registration number CAAE: 57024722.6.0000.5547.

This study was conducted in accordance with the ethical principles mentioned in the Declaration of Helsinki (2013).

The patient in this study consented to participate.

## Credit author statement

Celso Junio Aguiar Mendonça: Conceptualization, Investigation, Methodology, Project Administration, Writing - Original Draft, Writing – Review and Editing.

Gabriel Olesczuk: Formal Analysis.

Matheus Antunes Fernandes: Formal Analysis.

Ana Paula Ferreira: Formal Analysis, Methodology, Lucas Freitas Berti: Formal Analysis.

Ivan Moura Belo: Formal Analysis, Methodology, Jamil Faissal Soni: Conceptualization, Bertoldo Schneider Jr.: Conceptualization, Supervision, Writing – Review and Editing.

We declare that all authors have no conflicts of interest regarding this study.

Best Regards, Celso Júnio Aguiar Mendonça, MD, MSc, PhD.

## Funding statement

The authors declare that there has been no funding from any research agency for the current project.
